# Supplementary material for: Daily steps offset risks of sedentary behavior in the All of Us research program
Source: Nat Commun. 2026 Apr 7;17:4936. doi: 10.1038/s41467-026-71652-0 (PMC13234009; doi:10.1038/s41467-026-71652-0)

SUPPLEMENTARY MATERIALS

Supplementary Table 1. Hazard ratios and 95% confidence intervals comparing 75th percentile to 25th percentile derived from time-varying Cox proportional hazard models including sedentary cadence time only vs. sedentary cadence time and steps.

		Model with Sedentary Cadence Time	Model with Sedentary Cadence Time and Steps	
**Chronic Disease Phenotype**	**Case / Control**	**Sedentary Cadence Time HR (95% CI)**	**Sedentary Cadence Time HR (95% CI)**	**Steps HR (95% CI)**
Obesity	1281 / 6905	1.29 (1.23-1.36)	1.00 (0.94-1.06)	0.42 (0.38-0.47)
Diabetes Mellitus	170 / 11573	1.60 (1.39-1.85)	1.23 (1.05-1.44)	0.46 (0.35-0.60)
Hypertension	1171 / 5071	1.17 (1.12-1.22)	1.04 (0.98-1.09)	0.72 (0.67-0.78)
Atrial Fibrillation	198 / 12022	1.27 (1.13-1.43)	1.07 (0.93-1.24)	0.59 (0.46-0.75)
Heart failure	114 / 12286	1.68 (1.46-1.93)	1.38 (1.17-1.62)	0.52 (0.39-0.71)
CAD	382 / 11690	1.19 (1.08-1.32)	1.09 (0.97-1.24)	0.77 (0.63-0.93)
Ischemic Stroke	84 / 12333	1.07 (0.85-1.35)	0.89 (0.67-1.18)	0.54 (0.34-0.84)
CKD	235 / 12076	1.18 (1.04-1.34)	1.09 (0.94-1.27)	0.79 (0.62-1.01)
MASLD	478 / 12232	1.34 (1.22-1.48)	1.09 (0.97-1.23)	0.53 (0.43-0.65)
COPD	31 / 12023	1.61 (1.01-2.56)	1.14 (0.69-1.86)	0.46 (0.27-0.79)
MDD	591 / 9850	1.32 (1.22-1.42)	1.16 (1.06-1.27)	0.68 (0.59-0.78)
Sleep Apnea	849 / 11236	1.29 (1.21-1.38)	1.05 (0.97-1.14)	0.54 (0.48-0.62)

All Cox proportional hazards models were adjusted for age, sex, smoking status, alcohol drinking status, education status, and monthly averages of daily Fitbit wear time. Abbreviations: HR = hazard ratio; CI = confidence interval; CAD = coronary artery disease; CKD = chronic kidney disease; MASLD = metabolic dysfunction-associated steatotic liver disease; COPD = chronic obstructive pulmonary disease; MDD = major depressive disorder.

Supplementary Table 2. List of International Classification (ICD) billing codes, Current Procedural Terminology (CPT) procedure codes, and medications used to define chronic disease phenotypes for the study.

Condition	ICD-9 Codes	ICD-10 Codes	CPT Codes	Medications
**Atrial Fibrillation**	427.3, 427.31, 427.32	I48, I48.0, I48.1, I48.2, I48.3, I48.4, I48.9, I48.91, I48.92		
**Chronic Kidney Disease**	585, 585.%, 792.5, 996.73	V45%, V56%, N18%		
**Chronic Obstructive Pulmonary Disease**	491, 491.0, 491.1, 491.2, 491.20, 491.21, 491.22, 491.8, 491.9, 492, 492.0, 492.8, 496, 496.0, 493.21, 493.22	J44.%, J43.%, J42, J41.%		aclidinium, arformoterol, budesonide, ciclesonide, formoterol, glycopyrrolate, indacaterol, ipratropium, levalbuterol, olodaterol, revefenacin, roflumilast, salmeterol, tiotropium, umeclidinium

**Coronary Artery Disease**	410, 410.%, 411, 411.%, 412, 412.%, 413, 413.%, 414, 414.%, V45.82	I21, I21.%, I22, I22.%, I23, I23.%, I24, I24.%, I25, I25.%	33534, 33535, 33536, 33510, 33511, 33512, 33513, 33514, 33515, 33516, 33517, 33518, 33519, 33520, 33521, 33522, 33523, 92980, 92981, 92982, 92984, 92995, 92996	
**Diabetes Mellitus**	250.00, 250.02, 250.20, 250.22, 250.30, 250.32, 250.40, 250.42, 250.50, 250.52, 250.60, 250.62, 250.70, 250.72, 250.80, 250.82, 250.90, 250.92	E11.9, E11.65, E11.01, E11.00, E11.641, E11.29, E11.21, E11.319, E11.39, E11.36, E11.311, E11.40, E11.51, E11.630, E11.638, E11.620, E11.622, E11.621, E11.618, E11.628, E11.69, E11.649, E11.8		acetohexamide, tolazamide, chlorpropamide, glipizide, glyburide, glimepiride, repaglinide, nateglinide, metformin, rosiglitazone, pioglitazone, troglitazone, acarbose, miglitol, sitagliptin, exenatide, saxagliptin, linagliptin, liraglutide, semaglutide, canagliflozin, dapagliflozin, empagliflozin, alogliptin, colesevelam, albiglutide, dulaglutide, lixisenatide
**Heart Failure**	425, 425.%, 428, 428.%	I42, I42.%, I50, I50.%		

**Hypertension**			acebutolol, atenolol, betaxolol, bisoprolol, carvedilol, esmolol, labetalol, metoprolol, nadolol, nebivolol, pindolol, propranolol, sotalol, timolol, acetazolamide, dichlorphenamide, methazolamide, aliskiren, amiloride, eplerenone, finerenone, spironolactone, triamterene, amlodipine, clevidipine, diltiazem, felodipine, isradipine, nicardipine, nifedipine, nimodipine, nisoldipine, verapamil, azilsartan, candesartan, eprosartan, irbesartan, losartan, olmesartan, telmisartan, valsartan, benazepril, captopril, enalapril, enalaprilat, fosinopril, lisinopril, moexipril, perindopril, quinapril, ramipril, trandolapril, bumetanide, ethacrynic acid, furosemide, torsemide, chlorothiazide, chlorthalidone, hydrochlorothiazide, indapamide, metolazone, clonidine, guanabenz, guanfacine, hydralazine, lofexidine, mecamylamine, methyldopa, methyldopate, metyrosine, minoxidil
**Ischemic Stroke**	433.01, 433.11, 433.21, 433.31, 433.81, 433.91, 434.01, 434.11, 434.91, 436.%	I63.%, G46.3, G46.4	

**Major Depressive Disorder**	296.21, 296.22, 296.23, 296.24, 296.25, 296.26, 296.2, 296.31, 296.32, 296.33, 296.34, 296.35, 296.36, 296.3, 300.4, 293.83, 311	F32.0, F32.1, F32.2, F32.3, F32.4, F32.5, F32.9, F33, F33.1, F33.2, F33.3, F33.41, F33.42, F33.9, F34.1, F06.30, F32.9
**MASLD**	571.5, 571.8, 571.9	K74.0, K74.1, K74.2, K74.6%, K75.81, K76.0, K76.8, K76.89
**Sleep Apnea**	780.51, 780.53, 780.57, 327.2%	G47.3%

Supplementary Figure 1. Flow chart showing inclusion and exclusion criteria as well as the steps taken to clean the Fitbit data.

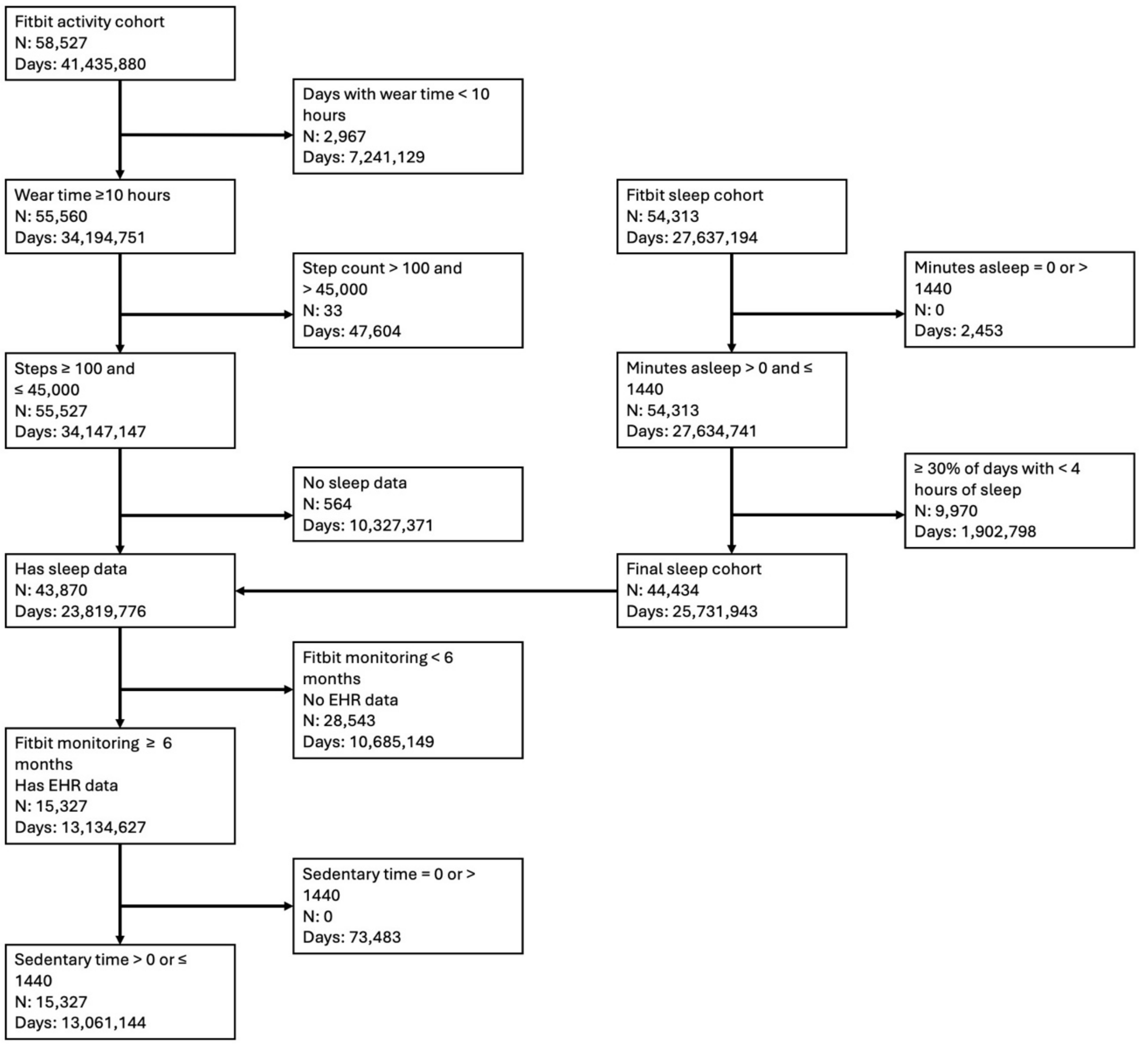

Supplementary Figure 2. Cumulative 3-year incidence of chronic diseases. Cox proportional hazard models were used to estimate cumulative 3-year incidence of disease as a function of average daily steps and stratified by different levels of sedentary time. All Cox proportional hazard models were adjusted for age, sex, smoking status, alcohol drinking status, and education status. Abbreviations: CAD = coronary artery disease; CKD = chronic kidney disease; MASLD = metabolic dysfunction-associated steatotic liver disease; COPD = chronic obstructive pulmonary disease; MDD = major depressive disorder.

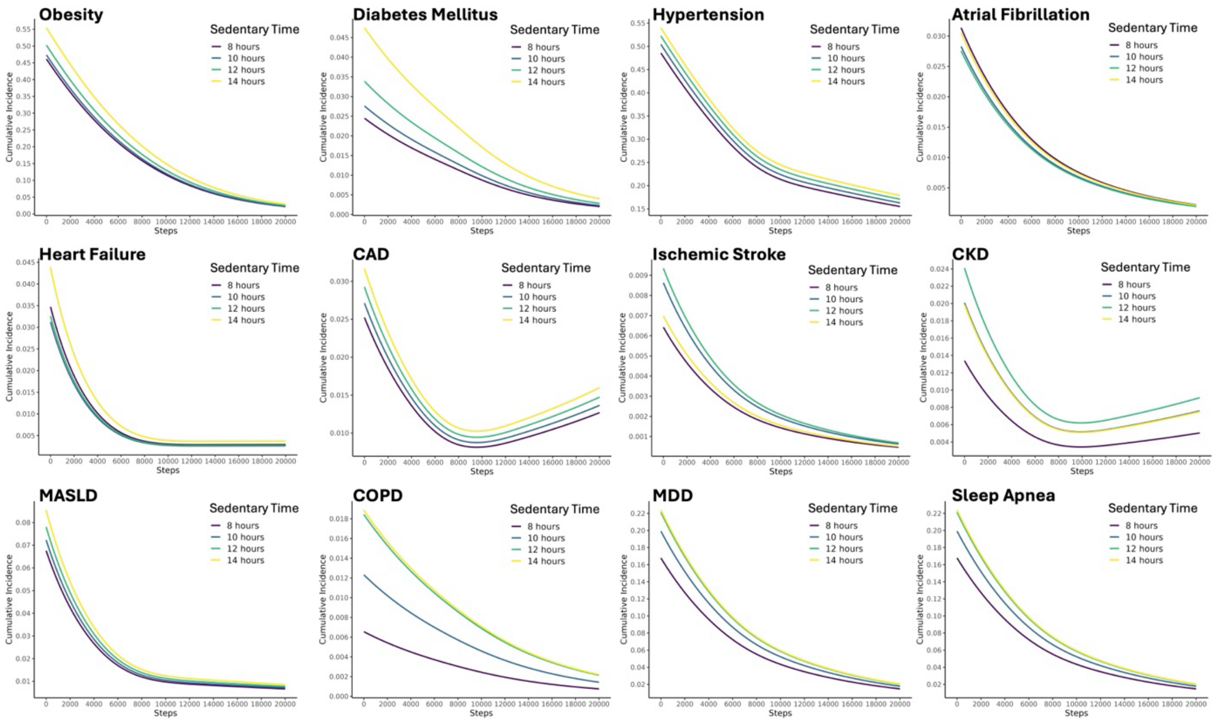

Supplement: Supplementary file 1 — Supplementary Information [file 41467_2026_71652_MOESM1_ESM.pdf]
