# Supplementary material for: Daily steps offset risks of sedentary behavior in the All of Us research program
Source: Nat Commun. 2026 Apr 7;17:4936. doi: 10.1038/s41467-026-71652-0 (PMC13234009; doi:10.1038/s41467-026-71652-0)
Supplement: Supplementary file 2 — Reporting Summary [file 41467_2026_71652_MOESM2_ESM.pdf]

nature portfolio

Corresponding author(s): Evan L. Brittain
Last updated by author(s): Feb 2, 2026

Reporting Summary

Nature Portfolio wishes to improve the reproducibility of the work that we publish. This form provides structure for consistency and transparency in reporting. For further information on Nature Portfolio policies, see our Editorial Policies and the Editorial Policy Checklist.

Statistics

For all statistical analyses, confirm that the following items are present in the figure legend, table legend, main text, or Methods section.

n/a	Confirmed	
☐	☒	The exact sample size (n) for each experimental group/condition, given as a discrete number and unit of measurement
☐	☒	A statement on whether measurements were taken from distinct samples or whether the same sample was measured repeatedly
☐	☒	The statistical test(s) used AND whether they are one- or two-sided<br>*Only common tests should be described solely by name; describe more complex techniques in the Methods section.*
☐	☒	A description of all covariates tested
☐	☒	A description of any assumptions or corrections, such as tests of normality and adjustment for multiple comparisons
☐	☒	A full description of the statistical parameters including central tendency (e.g. means) or other basic estimates (e.g. regression coefficient) AND variation (e.g. standard deviation) or associated estimates of uncertainty (e.g. confidence intervals)
☐	☒	For null hypothesis testing, the test statistic (e.g. F, t, r) with confidence intervals, effect sizes, degrees of freedom and P value noted<br>*Give P values as exact values whenever suitable.*
☒	☐	For Bayesian analysis, information on the choice of priors and Markov chain Monte Carlo settings
☒	☐	For hierarchical and complex designs, identification of the appropriate level for tests and full reporting of outcomes
☒	☐	Estimates of effect sizes (e.g. Cohen's d, Pearson's r), indicating how they were calculated

Our web collection on statistics for biologists contains articles on many of the points above.

Software and code

Policy information about availability of computer code

Data collection	No software was used for data collection.
Data analysis	To ensure participant's privacy, data from All of Us Research program can be accessed and analyzed through a secured cloud-based platform - Researcher Workbench. Statistical analyses were performed in R programming language (version 4.2.2, R Project https://www.r-project.org). Code used for this study can be made immediately available to any approved researchers on the All of Us Research Workbench platform by contacting our study team.

For manuscripts utilizing custom algorithms or software that are central to the research but not yet described in published literature, software must be made available to editors and reviewers. We strongly encourage code deposition in a community repository (e.g. GitHub). See the Nature Portfolio guidelines for submitting code & software for further information.

Data

Policy information about availability of data

All manuscripts must include a data availability statement. This statement should provide the following information, where applicable:

- Accession codes, unique identifiers, or web links for publicly available datasets
- A description of any restrictions on data availability
- For clinical datasets or third party data, please ensure that the statement adheres to our policy

To ensure privacy of participants, data used for this study are available to approved researchers following registration, completion of ethics training and attestation of a data use agreement through the All of Us Research Workbench platform, which can be accessed via https://workbench.researchallofus.org/.

Research involving human participants, their data, or biological material

Policy information about studies with human participants or human data. See also policy information about sex, gender (identity/presentation), and sexual orientation and race, ethnicity and racism.

Reporting on sex and gender	Self-reported sex was collected via The Basics survey by the All of Us research program. Self-reported sex was included as a covariate in all analyses.
Reporting on race, ethnicity, or other socially relevant groupings	Self-reported race was collected via The Basics survey by the All of Us research program and further categorized into White, Black or African American, Other, and Unknown. Self-reported race was included as a covariate in all analyses but was not used as a proxy for socioeconomic status.
Population characteristics	See below in "Behavioral & social sciences study design" section.
Recruitment	The All of Us research program (AoU) is a National Institutes of Health-funded initiative to gather health data from over one million diverse persons living in the United States (U.S.). Participants who provide consent and enrolled in the program to share multiple sources of health-related information, including electronic health records (EHRs), genomics, physical measures, and participant surveys. Participants who owned Fitbit devices have the opportunity to voluntarily share their Fitbit data under the Bring Your Own Device project, including physical activity and sleep patterns.
Ethics oversight	The All of Us research program's institutional review board has reviewed the protocol, informed consent, and other participant-facing materials for the All of Us Research Program. The IRB follows the regulations and guidance of the Office for Human Research Protections for all studies, ensuring that the rights and welfare of research participants are overseen and protected uniformly. To ensure privacy of participants, data used for this study was accessed and available to approved researchers only following registration, completion of ethics training, and attestation of a data use agreement through the All of Us Research Workbench platform, which can be accessed via https://workbench.researchallofus.org/login.

Note that full information on the approval of the study protocol must also be provided in the manuscript.

Field-specific reporting

Please select the one below that is the best fit for your research. If you are not sure, read the appropriate sections before making your selection.

☐ Life sciences ☒ Behavioural & social sciences ☐ Ecological, evolutionary & environmental sciences

For a reference copy of the document with all sections, see nature.com/documents/nr-reporting-summary-flat.pdf

Behavioural & social sciences study design

All studies must disclose on these points even when the disclosure is negative.

Study description	Observational longitudinal cohort study of quantitative data.
Research sample	15,327 adult participants with linked EHR data and sleep data met inclusion criteria for analysis, resulting in a total of 13,682,755 days of observation (eFigure 1). The median age was 51.7 years (interquartile range [IQR]: 37.3-63.5). Most participants were female (72.0%) and White (79.7%).
Sampling strategy	All of Us Research Program is an initiative that is accumulating multiple streams of health-related information (e.g., electronic health records (EHRs), genomics, physical measures, participant surveys and wearables such as Fitbit) in 1,000,000 or more Americans and includes a focus on populations usually under-represented in biomedical research to date. We used a convenience sampling procedure because our analysis was limited to the All of Us dataset. To determine the sample size, we followed the convention of having at least 10 events per predictor.
Data collection	All study participants consented and enrolled in the All of Us research program from May 2018 to October 2023, as described previously. We used data from the All of Us Controlled Tier Dataset v8 (C2024Q3R4), which is a retrospective electronic health record-based database available on the All of Us Researcher Workbench.
Timing	For this study, data from participants enrolled from May, 2017 to October, 2023 in All of Us Research Program were used.
Data exclusions	From over 633,000 All of Us participants enrolled at the time of analysis, 58,527 adult (≥18 years) participants voluntarily shared Fitbit device data through the Bring Your Own Device program or the Wearables Enhancing All of Us Research (WEAR) Study. Among the 58,527 individuals with Fitbit activity data, 15,327 adult participants with linked EHR data and sleep data met inclusion criteria for analysis. We excluded patients without Fitbit-derived sleep data to distinguish between sedentary time and sleep time.
Non-participation	We are using data from All of Us Research Program. As noted in the All of Us Responsible Conduct of Research training, the Researcher Workbench employs a data passport model, through which we are not conducting human subjects research with All of Us data for two reasons:(1) The research will not directly involve participants, however, it only includes their data; and (2) the data available in the Researcher Workbench has been carefully checked and altered to remove identifying information while preserving its scientific utility. Therefore, we do not have access to information around non-participation to the program.
Randomization	No randomization was performed for this study.

Reporting for specific materials, systems and methods

We require information from authors about some types of materials, experimental systems and methods used in many studies. Here, indicate whether each material, system or method listed is relevant to your study. If you are not sure if a list item applies to your research, read the appropriate section before selecting a response.

Materials & experimental systems

n/a	Involved in the study
☒	☐ Antibodies
☒	☐ Eukaryotic cell lines
☒	☐ Palaeontology and archaeology
☒	☐ Animals and other organisms
☒	☐ Clinical data
☒	☐ Dual use research of concern
☒	☐ Plants

Methods

n/a	Involved in the study
☒	☐ ChIP-seq
☒	☐ Flow cytometry
☒	☐ MRI-based neuroimaging

Plants

Seed stocks	N/A
Novel plant genotypes	N/A
Authentication	N/A
